# Anti-aging effect of β-carotene through regulating the KAT7-P15 signaling axis, inflammation and oxidative stress process

**DOI:** 10.1186/s11658-022-00389-7

**Published:** 2022-10-08

**Authors:** Wei V. Zheng, Wang Xu, Yaqin Li, Jie Qin, Tao Zhou, Dezhi Li, Yanwei Xu, Xianyi Cheng, Yu Xiong, Zaizhong Chen

**Affiliations:** 1 Intervention and Cell Therapy Center, Peking University Shenzhen Hospital, Shenzhen, Guangdong 518036 People’s Republic of China; 2 Department of Minimally Invasion Intervention, Peking University Shenzhen Hospital, Shenzhen, Guangdong 518036 People’s Republic of China; 3 Department of Infectious Disease, Peking University Shenzhen Hospital, Shenzhen, Guangdong 518036 People’s Republic of China; 4 College of Veterinary Medicine, Northwest A&F University, Yangling, 712100 China; 5 Scientific and Resaerch Dept., Peking University Shenzhen Hospital, Shenzhen, Guangdong 518036 People’s Republic of China

**Keywords:** β-Carotene, Aging, Stem cells, KAT7-P15 signaling axis

## Abstract

**Background:**

Research on aging is growing as the elderly make up a greater share of the population, focusing on reversing and inhibiting the aging process. The exhaustion and senescence of stem cells are the fundamental drivers behind aging. β-Carotene has been depicted to have many biological functions, and we speculate that it may have an anti-aging effect.

**Methods:**

Firstly, the anti-aging property of β-carotene was investigated in vitro using mesenchymal stem cells (MSCs) induced by H_2_O_2_. The anti-aging effect was characterized using Western-bloting, confocal laser scanning microscopy, indirect immunofluorescence, and immunohistochemistry. The anti-aging property was also tested in vivo using aged mice.

**Results:**

The in vitro experiment revealed that β-carotene could relieve the aging of MSCs, as evidenced by a series of aging marker molecules such as p16 and p21. β-Carotene appeared to inhibit aging by regulating the KAT7-P15 signaling axis. The in vivo experiment revealed that β-carotene treatment has significantly down-regulated the aging level of tissues and organs.

**Conclusions:**

In this work, we explored the anti-aging effect of β-carotene in vivo and in vitro. The experimental results indicate that β-carotene may be an important potential anti-aging molecule, which can be used as a drug or in functional food to treat aging in the future.

**Supplementary Information:**

The online version contains supplementary material available at 10.1186/s11658-022-00389-7.

## Introduction

As the average age of the human population trends upwards, research on aging has gradually become a center of attention. Aging refers to the inevitable functional decline of an organism's organs at the end of its lifespan, accompanied by a lowered capacity to stabilize its internal environment and deal with stress [1, 2]. Cell senescence is a prerequisite of body aging. Studies have revealed that adult stem cells (such as Mesenchymal stem cells) play an important role in maintaining body health and preventing age-related diseases [3, 4]. Cell senescence can be categorized depending on its inducing factor into stress-induced premature senescence (SIPS), replicative senescence (RS), drug-induced senescence and oncogene-induced aging [5]. Cell senescence is characterized by morphological changes, cell cycle arrest, oxidative stress, and chromatin changes [6]^.^

Adult stem cells (adipose mesenchymal stem cells, bone marrow mesenchymal stem cells, umbilical cord mesenchymal stem cells) can self-renew and differentiate into many kinds of cells [7]. MSCs undergo aging when stimulated by various factors, leading to significantly weakened proliferation and differentiation of senescent stem cells. Researchers have explored many approaches to prevent stem cell aging. The emergence of high-throughput sequencing technology has led to the identification of a series of genes related to aging [8]. Anti-aging drugs have been discovered, such as metformin [9]. The same effect has also been identified in plant and animal extracts, such as NAD^+^ [10].

Anti-aging therapy is a long-term engagement since the body would continue to age otherwise. Therefore, naturally-sourced and healthy nutritional compounds are preferred in anti-aging therapy. β-Carotene is a carotenoid, a natural pigment, and a micronutrient with many biological functions. It is an antioxidant that has been depicted to inhibit the incidence and development of cancer. It also has an anti-inflammatory effect in various animal and cell models [11].

Based on the physicochemical nature of β-carotene, we speculated that it might have an anti-aging effect. To test this hypothesis, an aging model of MSCs induced by H_2_O_2_ was established. The anti-aging effect of β-carotene was evaluated through a series of aging marker molecules in vitro. We also investigated the anti-aging effect in vivo*.* Current research reveals that β-carotene may be an important anti-aging molecule, which means that it can be administered as a supplement or an ingredient in functional food to relieve aging.

## Materials and methods

### Antibodies and reagents

β-Carotene was purchased from Sigma-Aldrich (Cat.no.7235-40-7). CD73 (ab202122, 1:200 dilution), CD90 (ab23894, 1:500 dilution), CD105 (ab2529, 1:200), P16 (ab220800, 1:1000), and P15 (ab53034, 1:500 dilution) were purchased from Abcam (United Kingdom). DMEM/F-12, fetal bovine serum (FBS), and non-essential amino acids (NEAA) were purchased from Thermo Fisher. Cell cycle and apoptosis detection kit and cell aging β-Galactosidase staining kit were purchased from Biyuntian (Shanghai, China). DNA extraction kit was purchased from Tiangen; Osteogenic induction differentiation medium and adipogenic induction differentiation kit were purchased from Thermo Fisher. ROS detection kit was purchased from Sigma-Aldrich. CCK-8 kit was purchased from Dojindo Laboratories (Kumamoto, Japan).

### Cell culture

MSCs were purchased from Thermo Fisher (Cat.no.MSCA15652). They were stored in our laboratory. The MSCs (passage 3) were seeded into a gelatin-coated 6-well plate at a density of 2 × 10^4^/cm^2^ and cultured in 10% FBS DMEM at 37 °C.

### Establishment of MSCs aging model

In this work, we constructed the senescent MSC model using H_2_O_2_ as the aging agent [9, 13]. In the preliminary screening, MSCs were treated with various concentrations of H_2_O_2_ from 0 to 100 μM. When the appropriate hydrogen peroxide dose had been determined, the MSC aging model was constructed. The cells were cultured for 48 h, after which their senescence was assessed using the Sa-β-gal staining kit.

### Cell proliferation analysis

CCK8 detection kit was used to detect the effect of β-carotene on the proliferation of MSCs. MSCs were digested and prepared into a cell suspension with cell density adjusted to 5 × 10^4^/mL. A 100 μL cell suspension volume was added to a 96-well plate and incubated in a CO_2_ incubator overnight. The medium was discarded, different concentrations of β-carotene were added, and the cells were incubated at 37 °C for 24 h. The drug culture medium was discarded, and CCK8 solutions were added. The plate was again incubated in CO_2_ incubator for 1 h. The cell samples were analyzed in a spectrophotometer at 450 nm.

### Cell transfection

KAT7 and P15 cDNA were kindly provided by Hua-Cheng Medical and Biological Co. Ltd. KAT7 and P15 cDNAs were cloned into the mammalian expression vector pCDNA3.1 according to the manufacturer's instructions. MSCs were transfected with pcDNA3.1 KAT7 and P15 cDNAs or an empty vector (as a control) using Lipofectamine 3000 following the manufacturer's instructions. IFA and Western blot tests were used to evaluate transfection efficiency.

### Adipogenic differentiation of MSCs

The cell culture medium was discarded. The cell culture was washed twice with phosphate-buffered saline (PBS), then fixed using 4% formaldehyde (PFA) solution for 30 min. The fixed cells were washed with PBS and stained with 12 mL of oil red staining solution for 30 min. The stained cells were rinsed thrice and were observed and imaged under a microscope.

### Senescence-associated (SA) β-galactosidase (β-gal) staining

After the cells on the culture plate had been treated with β-carotene, they were washed with PBS and fixed in 4% paraformaldehyde at room temperature for 15 min. The fixed cells were washed with PBS and incubated with fresh SA-β-Gal staining solution at 37 °C for 12 h. The SA-β-gal positive cells were observed and counted under a light microscope.

### ELISA assays

TNFα and IL6 levels were measured using an ELISA kit following the manufacturer's instructions.

### RT-PCR analysis

Following β-carotene treatment, the cells were digested with 0.25% trypsin, washed, and the supernatant was discarded. Total RNA was extracted using Trizol (1 mL) following the manufacturer's instructions. The total RNA was reverse transcribed into cDNA. RT-PCR was performed using the primers demonstrated in Additional file 1: Table S1.

### Western blot

After β-carotene treatment, the culture medium was discarded. After washing the cells with PBS thrice, the cells were lysed using RIP lysis buffer. The cell lysate was centrifuged at 14,000×*g* for 15 min at 4 °C. After centrifugation, the supernatant was collected. Protein concentrations were then determined using the BCA kit. The protein samples were heated at 98 °C for 5 min and then immediately placed on ice for 5 min. 30 μg/well (protein sample) was subjected to SDS-PAGE analysis, and then the protein samples were transferred to PVDF membrane. After washing, the cell samples were blocked with 5% BSA at room temperature for 2 h in a shaker. After washing, the PVDF membranes were incubated with the indicated primary antibodies for 12 h at 4 °C, followed by incubation with secondary antibodies at 37 °C for 1 h. After the final three washes, the ECL system was used to visualize protein blots.

### Indirect immunofluorescence assay

The cells were seeded on glass slides in 6-well culture plates. When the cells reached 50% confluence, they were fixed with 4% PFA at 37 °C for 10 min, then washed with PBS. The fixed cells were permeabilized with 0.1% Triton X-100 in PBS at room temperature for 15 min, then washed. The cells were sealed in 5% BSA for 30 min, then washed again. Primary antibody was added, and the cells were incubated at 4 °C for 12 h, after which they were washed thrice with PBS. The cells were incubated again with the secondary antibody (1:500) at room temperature for 1 h and then washed. The cells were stained with DAPI for 10 min, then imaged under a Confocal laser scanning microscope (Leica TCS SP8 STED) and analyzed with ImagePro Plus 6.0.

### Flow cytometry analysis

When the cells had grown to 80%, they were treated with β-carotene, then washed thrice. The cells were digested using 0.25% trypsin and collected via centrifugation. They were fixed in 4% PFA, then incubated with primary antibodies for 2 h at 37 °C, and stained with fluorescent labeled-secondary antibodies. Finally, cell samples were analyzed via Flow Cytometry (BD AccuriC6 Flow Cytometry).

### ROS detection

MSCs were digested into a cell suspension and counted. The cell density was adjusted to 5 × 10^4^/mL, and the suspension was transferred to a 96-well plate, 100 μL per well. The cell samples were incubated in a CO_2_ incubator overnight. β-Carotene medium with appropriate concentration was added. Cell samples were prepared and analyzed via flow cytometry.

### Animals and β-carotene administration

All animals were treated strictly following the guidelines of the Animal Care and Use Committee of Peking University Shenzhen Hospital. Thirty male C57 mice (22 months old, 25–30 g body weight) were purchased from Huafukang bioscience company (Beijing, China). They had free access to water in an air-conditioned room (22–25 °C) and lighting (12-h light-darkness cycle). The mice were divided into the β-carotene groups administered with micellar β-carotene and the vehicle group administered with only micellar. The mice were orally administered their respective treatment once daily, 0.5 mg each dose, via direct intubation to the stomach.

### Hematoxylin–eosin staining

The tissue samples were fixed, dehydrated and embedded in paraffin. The paraffin samples were cut into thin sections, 4 μM thick. The sections were re-hydrated in PBS for 5 min, stained with hematoxylin staining solution for 5 min, then washed with running water and observed under a microscope.

### Y-maze test

The mice were transferred from the animal room into the behavioral laboratory in advance to adapt to the experimental environment. The testing environment in the laboratory was kept quiet. Before starting the experiment, the mice were placed in the center of the Y-maze and shuttled through the three arms. The sequence of mice entering and leaving each arm within 5 min was recorded and analyzed using Shanghai XIN-RUAN Visutrack animal behavior analysis software. The correct choice was to enter three different arms continuously. The correct selection times "n" and total arm entry times "n" of each mouse were recorded, and the correctness rate of each mouse was calculated.

### Statistical analysis

The experimental results were expressed as mean ± standard deviation (SD) and were measured with graph pad prism 8.0. p-value was calculated via the t-test or one-way analysis of variance (ANOVA). p-value < 0.05 was considered statistically significant.

## Results

### Construction of senescence model of Ad-MSCs by H_2_O_2_ treatment (in vitro)

**Fig. 1:**
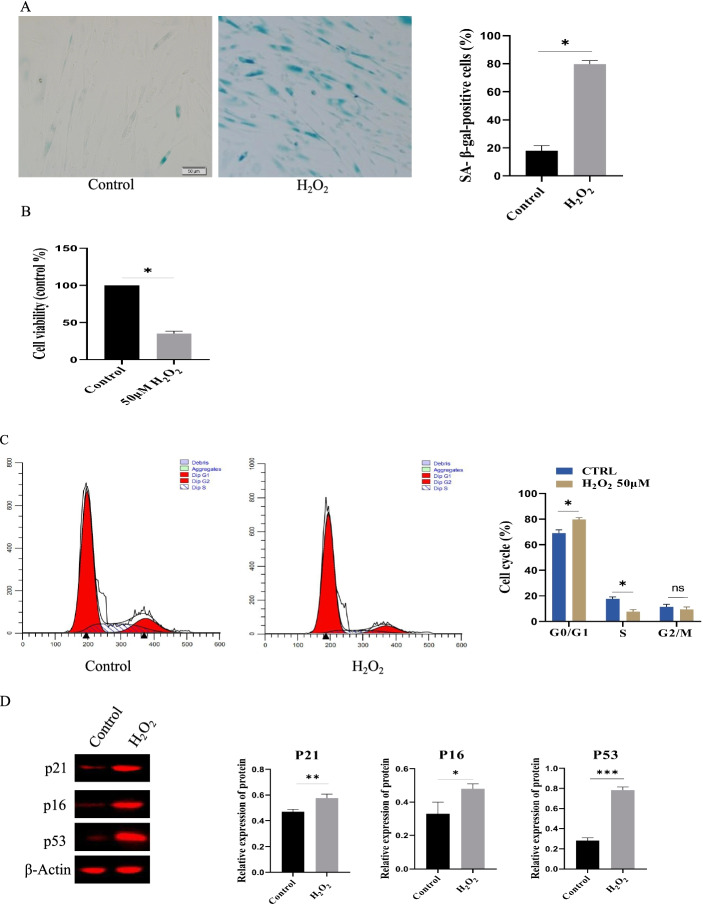
Establishment of Ad-MSCs senescence model by 50 μM H_2_O_2_ treatment. **A** Percentage of Sa-β-gal-positive cells were significantly decreased under H_2_O_2_ treatment, Scale bar, 20 μm. n = 5 biological replicates*.* **B** H_2_O_2_ (50 μM) treatment inhibited the cell proliferation of Ad-MSCs. **C** The effect of H_2_O_2_ (50 μM) treatment on cell cycle phase by Flow cytometry analyses, n = 3 wells per cell type. **D** The expression of P21, P16 and P53 was down-regulated under H_2_O_2_ treatment

The senescent Ad-MSC model was constructed using 50 μM H_2_O_2_ as the aging agent for 2 h of treatment, based on the finding from the preliminary screening. The Sa-β-gal-positive cell ratio in the 50 μM H_2_O_2_ treatment group was significantly improved than the control group (p < 0.05) (Fig. 1A). CCK8 revealed that cell proliferation was inhibited by H_2_O_2_ treatment than in the control group (p < 0.05) (Fig. 1B). The results from the cell cycle detection via flow cytometry indicated that the G0/G1 phase ratio significantly increased and the ratio of S-phase cell significantly decreased (control group: 19% versus 50 μM H_2_O_2_ group: 8%) (Fig. 1C). Additionally, the senescence-related markers, P16, P21, and P53, were obviously up-regulated in the H_2_O_2_ group (Fig. 1D).

**Fig. 2:**
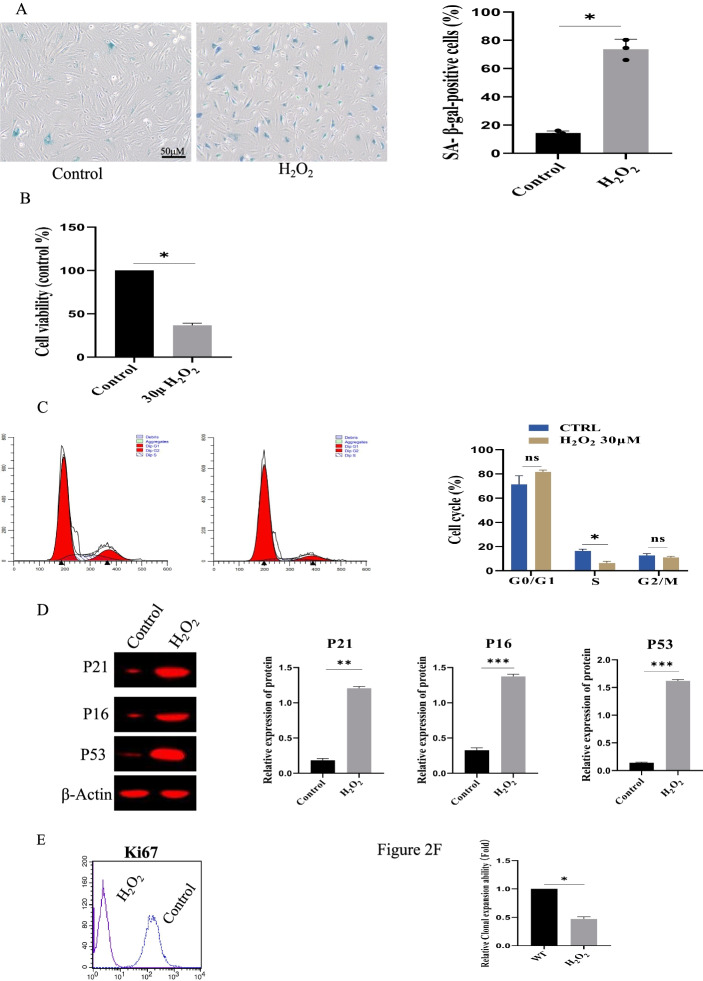
Establishment of Bm-MSCs senescence model by 30 μM H_2_O_2_ treatment. **A** The percentage of Sa-β-gal-positive cells were significantly decreased under H_2_O_2_ treatment. Scale bar, 20 μm. n = 5 biological replicates. **B** H_2_O_2_ treatment inhibited the cell proliferation of MSCs, n = 3. **C** The effect of H_2_O_2_ (30 μM) treatment on cell cycle phase by Flow cytometry analyses. **D** The expression of p21, p16 and p53 was down-regulated under H_2_O_2_ (n = 3). **E** The expression of Ki67 was down-regulated. At least 50 cells were statistically analyzed. **F** Clone expansion capability was reduced by H_2_O_2_ (n = 3)

The senescent bone marrow mesenchymal stem cell (Bm-MSC) model was successfully constructed using 30 μM H_2_O_2_ aging agent, based on the finding from the preliminary screening, as displayed in Fig. 2A. After treatment with 30 μM H_2_O_2_, Sa-β-gal-positive cell ratio was significantly improved (p < 0.05). CCK8 assays demonstrated that Bm-MSC proliferation was inhibited by 39% ± 8% than in the control group (Fig. 2B). Cell cycle analysis revealed that the G0/G1 phase ratio increased significantly, and the percentage of S-phase cells decreased (Fig. 2C). P16, P21, and P53 expressions were significantly reduced (Fig. 2D), the expression of Ki67 was significantly down-regulated (Fig. 2E), and the clone expansion capability was also reduced than the control group (Fig. 2F).

### β-Carotene alleviates the senescence of MSCs (in vitro)

**Fig. 3:**
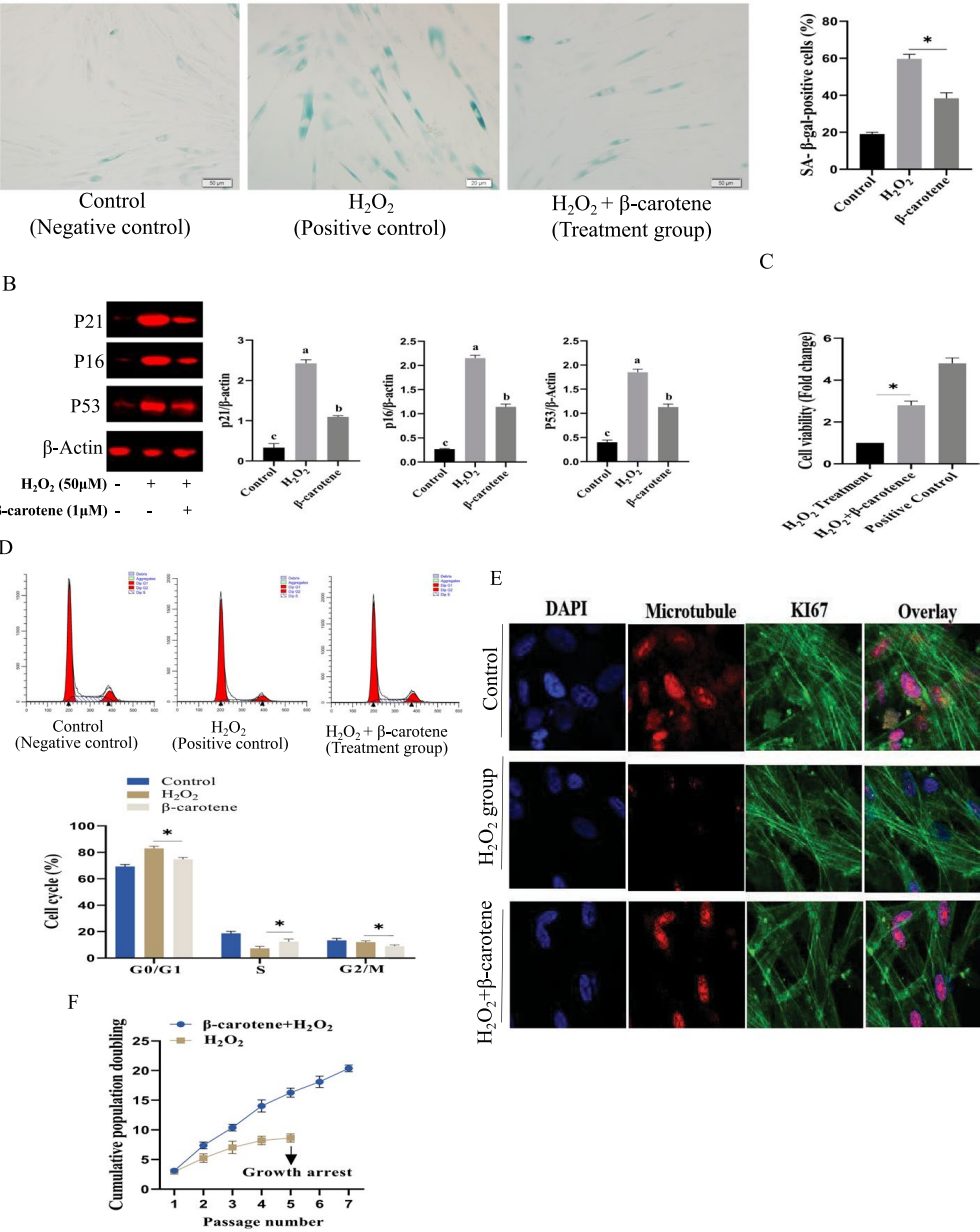
β-Carotene alleviated the Ad-MSCs aging. **A** Sa-β-gal positive cells were up-regulated under β-carotene treatment, n = 5 biological replicates. **B** The expression level of p16/p21/p53 was down-regulated under β-carotene treatment. **C** β-carotene enhanced the proliferation of Ad-MSCs, n = 3 biological replicates. **D** the effect of β-carotene on cell cycle (n = 3 wells per cell type). **E** β-carotene treatment up-regulated Ki67 expression. **F** β-carotene treatment increased the growth rate of Ad-MSCs.

The β-carotene treatment was found to alleviate the senescence of Ad-MSCs. The ratio of SA-β-gal-positive cells in the β-carotene treatment group was significantly reduced than the control group (p < 0.05) (Fig. 3A). The expression of P21, P16, and P53, were also down-regulated (Fig. 3B). The results of CCK8 assay demonstrated that the proliferation of Ad-MSCs was significantly enhanced than the control group (Fig. 3C). Cell cycle analyses also indicated that the S phase ratio of Ad-MSCs was significantly enhanced (Fig. 3D). The percentage of Ki67-positive stem cells was significantly increased than the control group (Fig. 3E). The effect of β-carotene on the growth rate of Ad-MSCs via serial passaging was also investigated, and the results demonstrated that β-carotene treatment enhanced the growth rate of Ad-MSCs (Fig. 3F).

**Fig. 4:**
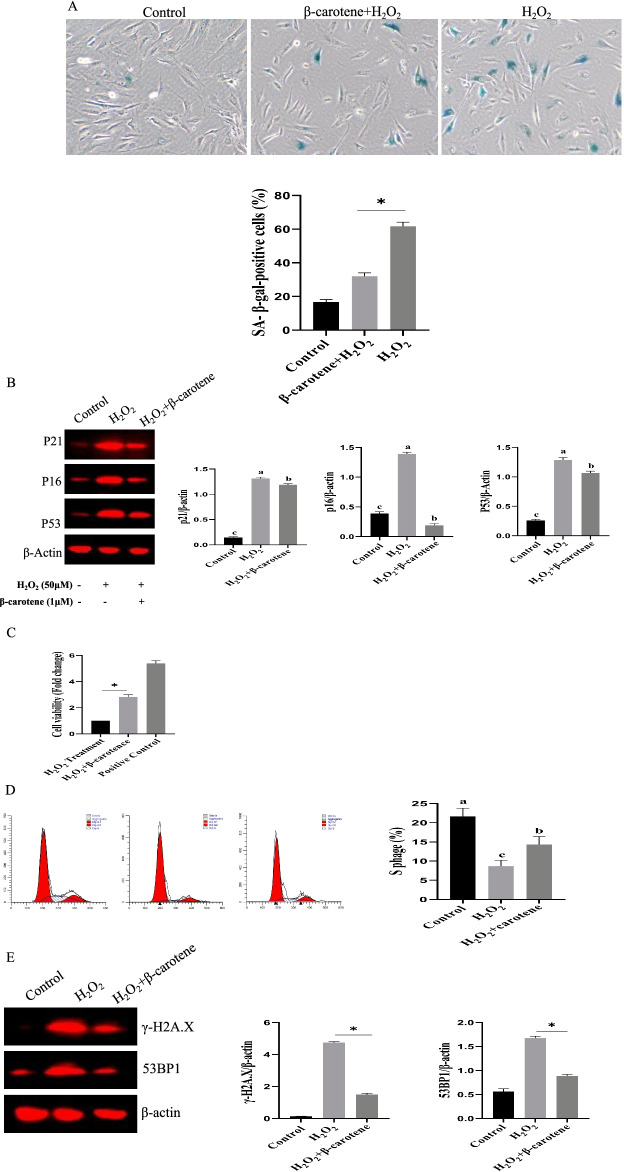
**A** β-carotene alleviated the aging of Bm-MSCs. **A** Sa-β-gal positive cells were decreased under β-carotene treatment. n = 5 biological replicates. **B** The expression levels of p16/p21/p53 was reduced under β-carotene treatment. **C** Bm-MSCs proliferation was increased under β-carotene treatment. n = 3 biological replicates. **D** Cell cycle was changed by β-carotene treatment. **E** β-carotene treatment reduced the expression of γ-H2A.X and 53BP1 expression

Additionally, we found that β-carotene could alleviate the aging of Bm-MSCs. The proportion of SA-β-gal positive cells decreased significantly in the β-carotene treatment group than in the control group (p < 0.05) (Fig. 4A). The expression of p21, p16, and p53 were also down-regulated by β-carotene (Fig. 4B). CCK8 assays revealed that the Bm-MSCs proliferation significantly increased than the control group (Fig. 4C). Cell cycle analyses indicated that the S phase ratio of Bm-MSCs was significantly enhanced (Fig. 4D). The expression of γ-H2A.X (a molecular marker of DNA damage response) was reduced (Fig. 4E). Furthermore, the key DNA damage response protein (53BP1) was downregulated at 53BP1.

### Effect of β-carotene on replicative senescence of Ad-MSCs (in vitro)

**Fig. 5:**
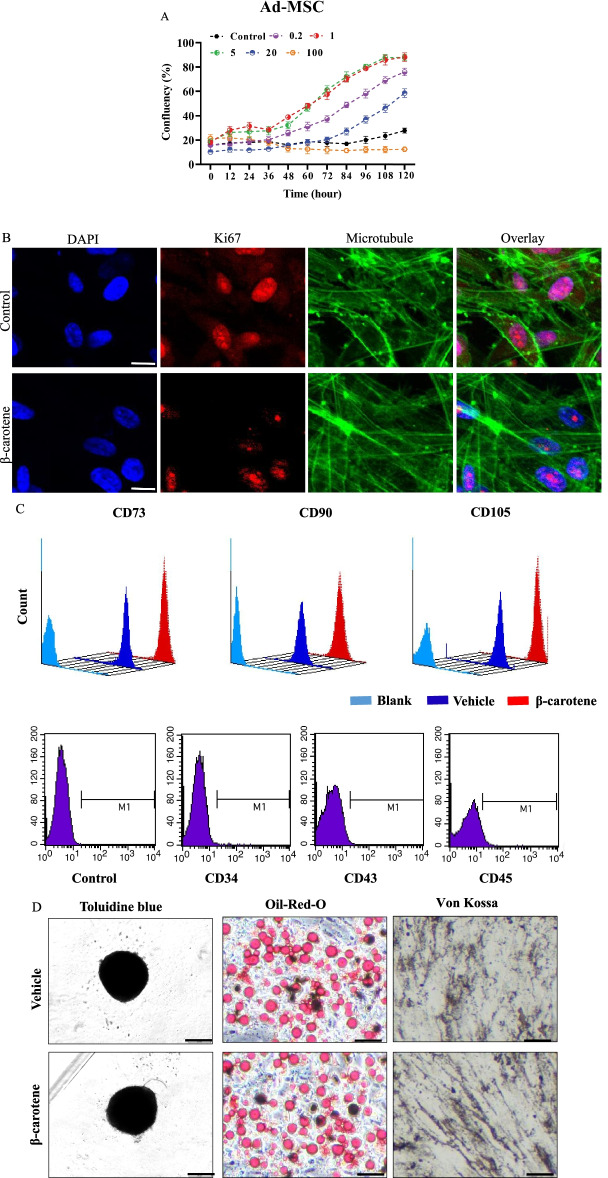
Effect of β-carotene on replicative senescence of Ad-MSCs. **A** Relative cell proliferation detection upon β-carotene treatment by using the IncuCyte S3 live-cell imaging system. n = 5 biological replicates. **B** The percentage of Ki67-positive cells was increased by β-carotene treatment. At least 50 cells were statistically analyzed. **C**, **D** Effect of β-carotene on the properties of MSCs

Firstly, we evaluated the effect of β-carotene on Ad-MSCs regeneration. The cells were treated with various concentrations of β-carotene, and the results indicated that β-carotene promoted MSCs self-renewal at lower concentrations (0.5–5 μM). At higher concentrations above 20 μM, β-carotene inhibited Ad-MSC proliferation (Fig. 5A). Furthermore, the 1 μM β-carotene treatment, which was within the physiological range, increased the percentage of Ki67-positive cells (Fig. 5B). Therefore, 1 μM β-carotene was used in the current study. To determine if cell properties were affected by β-carotene treatment, cell surface markers, cellular functions, and differentiation potential were analyzed. As illustrated in Fig. 5C, β-carotene treatment did not affect the markers of MSCs; FACS results revealed that Ad-MSCs were positive for typical MSC markers, such as CD73, CD90, and CD105, and negative for hMSC-irrelevant antigens, including CD34, CD43, and CD45. Additionally, β-carotene did not affect the differentiation ability of MSCs (Fig. 5D).

**Fig. 6:**
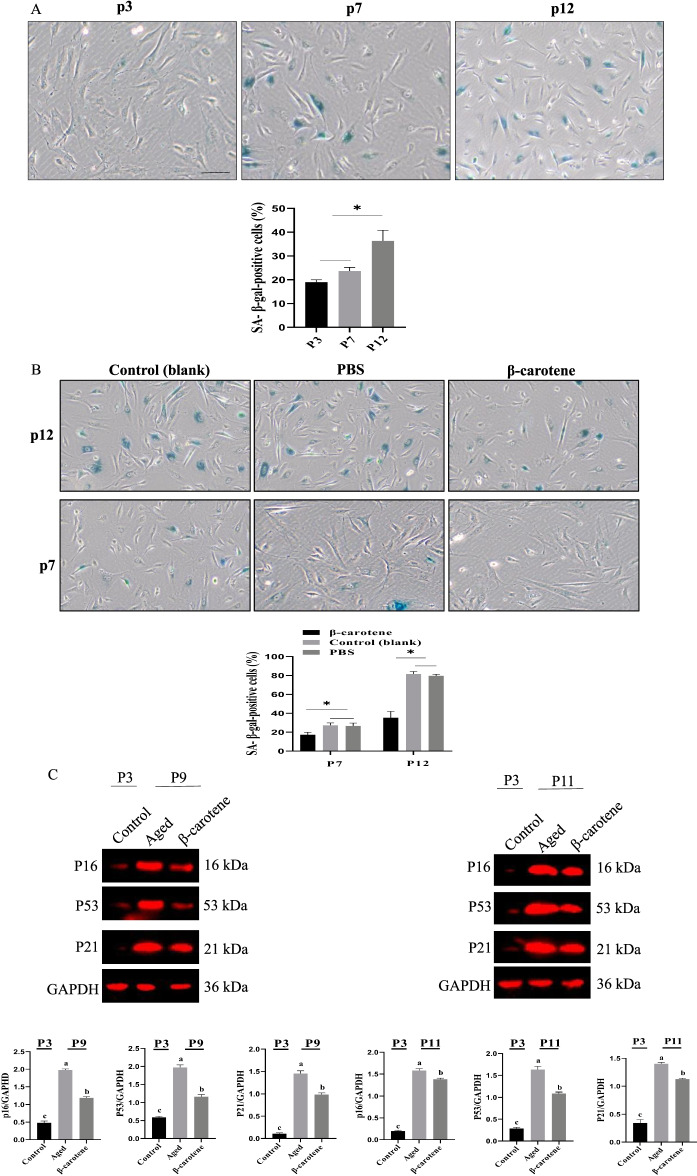

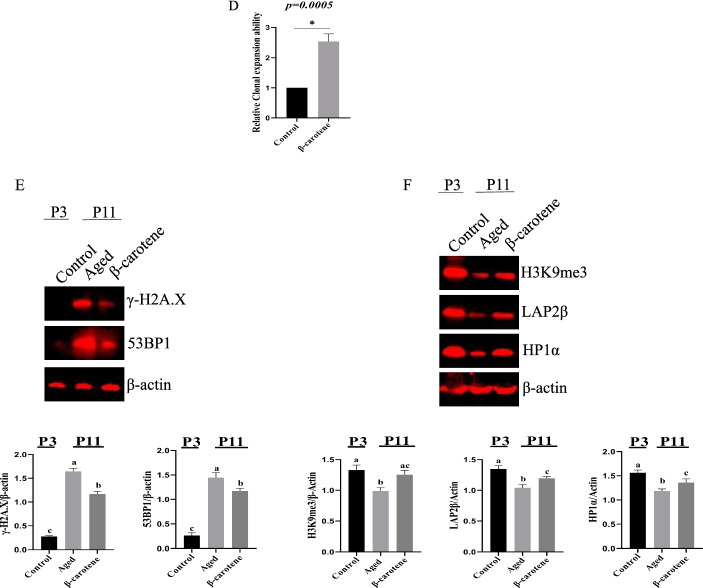
The effect of β-carotene on replicative aging in Ad-MSCs. **A** Analysis of aging of MSCs upon serial passage. n = 3 biological replicates. **B** The ratio of SA-β-gal-positive cells was down-regulated. n = 3 biological replicates. **C** The expression level of p16, p53, and p21 was down-regulated by β-carotene treatment. **D** β-carotene treatment increased the clonal expansion ability of MSCs. **E** H3K9me3 expression was increased by β-carotene treatment. **F** β-carotene treatment up-regulated HP1α and H3K9me3

Next, we analyzed the effect of β-carotene on replicative aging in Ad-MSCs. The percentage of SA-β-gal-positive Ad-MSCs was significantly increased with serial passage (Fig. 6A). However, the treatment with β-carotene partially reduced the ratio of SA-β-gal-positive cells (Fig. 6B). The expression level of p53, p16, and p21 in the MSCs with subculture were significantly enhanced with a cell passage. In contrast, β-carotene significantly reduced the expression level of p16/p53/p21 than the control group (Fig. 6C). Further analyses demonstrated that β-carotene treatment increased the clonal expansion ability of MSCs (Fig. 6D). 53BP1 and γ-H2A.X expressions were also down-regulated (Fig. 6E). In addition, the levels of heterochromatin markers (HP1α and H3K9me3) as determined via Western blotting demonstrated that β-carotene treatment up-regulated HP1α and H3K9me3, indicating that β-carotene promoted the remodeling of heterochromatin to a younger state (Fig. 6F).

### β-Carotene demonstrated anti-inflammatory effects on the aged MSCs (in vitro)

**Fig. 7:**
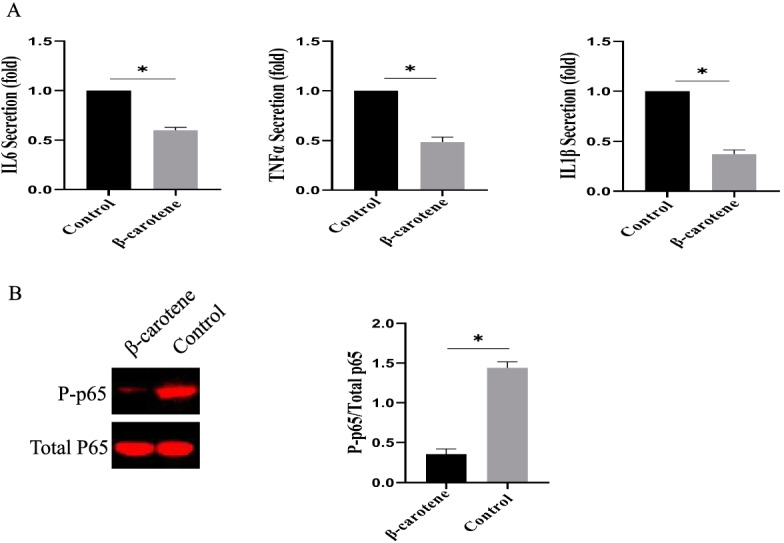
The effect of β-carotene on inflammation in vitro. **A** The effect of β-carotene on the aging-induced inflammation in Ad-MSCs and Bm-MSCs. n = 3 biological replicates. **B** The effect of β-carotene on NF-κb expression. Data are expressed as mean ± SD. The asterisk indicates a significant difference (p < 0.05)

Inflammation is also involved in the aging process of MSCs, which is one of the factors leading to aging [12]. Therefore, we analyzed the effect of β-carotene on inflammation in aged MSCs. In Fig. 7A, β-carotene significantly reduced the level of IL-1 β, IL-6, and TNF-α. The phosphorylation levels of NF-κb in the β-carotene treatment group were also significantly down-regulated (Fig. 7B).

### β-Carotene exhibited an antioxidant stress effect in MSCs (in vitro)

**Fig. 8:**
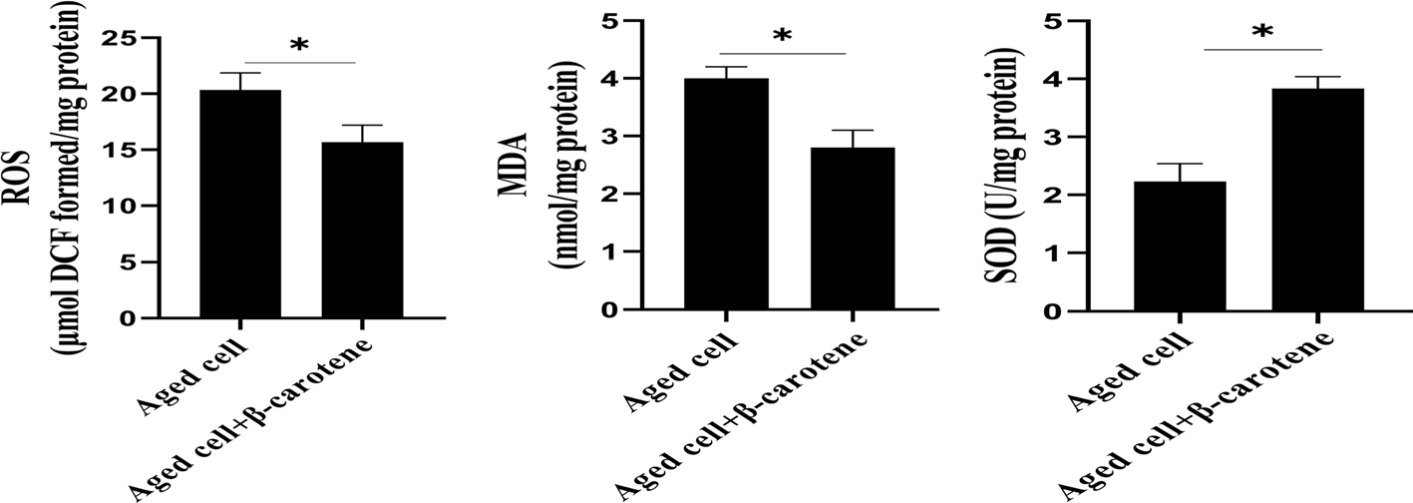
The effect of β-carotene on oxidative stress. The detailed process of the experiment has been described in the Materials and Methods section

Oxidative stress is one of the most important factors that promote the aging of MSCs [13]. For this, we evaluated the effect of β-carotene on the oxidative stress markers in MSCs. In Fig. 8, β-carotene pre-treatment significantly reduced the levels of ROS and MDA and increased the levels of SOD, indicating that β-carotene acted as an antioxidant.

### β-Carotene alleviated MSCs aging by regulating KAT7-P15 signaling (in vitro)

**Fig. 9:**
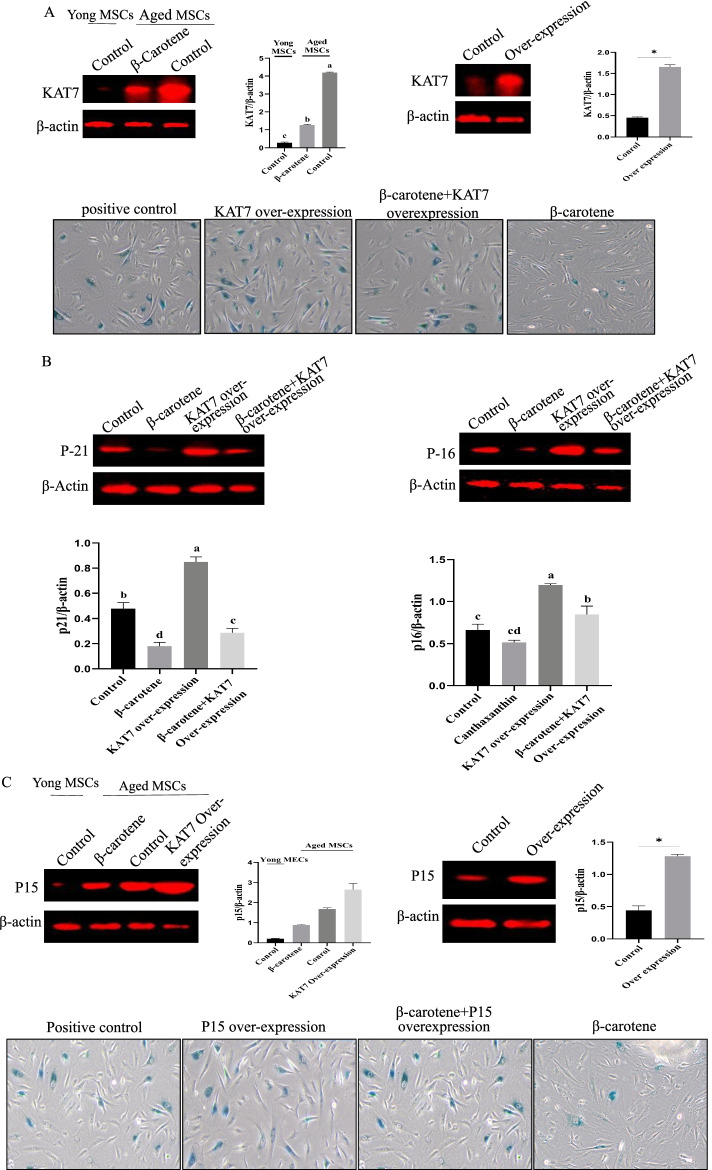
Analysis of the potential anti-aging mechanism of β-carotene. **A** The effect of β-carotene on KAT7 expression. n = 3 biological replicates. **B** Effect of KAT7 over-expression on β-carotene anti-aging. **C** Effect of p15 over-expression on β-carotene anti-aging

The finding that β-carotene was an antioxidant and an anti-inflammatory are good evidence that β-carotene could exhibit an anti-aging effect. With that said, there are other potential mechanisms by which β-carotene could relieve aging. Previous studies have found that MSC aging involves many molecules, including transcription factors [14]. For this, we conducted a preliminary screening and found that the expression of KAT7 was down-regulated after β-carotene treatment (Fig. 9A), and recent studies have displayed that KAT7-P15 axis is involved in the aging of MSCs. Based on this clue, we posited that β-carotene exhibited anti-aging properties (at least in part) by regulating the KAT7-P15 signaling axis. To test this hypothesis, we conducted a rescue experiment on the over-expression of KAT7. We found that ectopic expression of KAT7 partially offset the anti-aging effect of β-carotene (Fig. 9B). To further confirm whether β-carotene exhibits an anti-aging effect through the KAT7-P15 axis, we found that the ectopic expression of p15 partially neutralized the anti-aging effect of β-carotene (Fig. 9C). Furthermore, we found that the ectopic expression of KAT7 also up-regulated p15, which is similar to the results of previous studies, indicating that p15 acts downstream of KAT7 in mediating cellular senescence.

### β-Carotene demonstrated an anti-aging effect (in vivo)

**Fig. 10:**
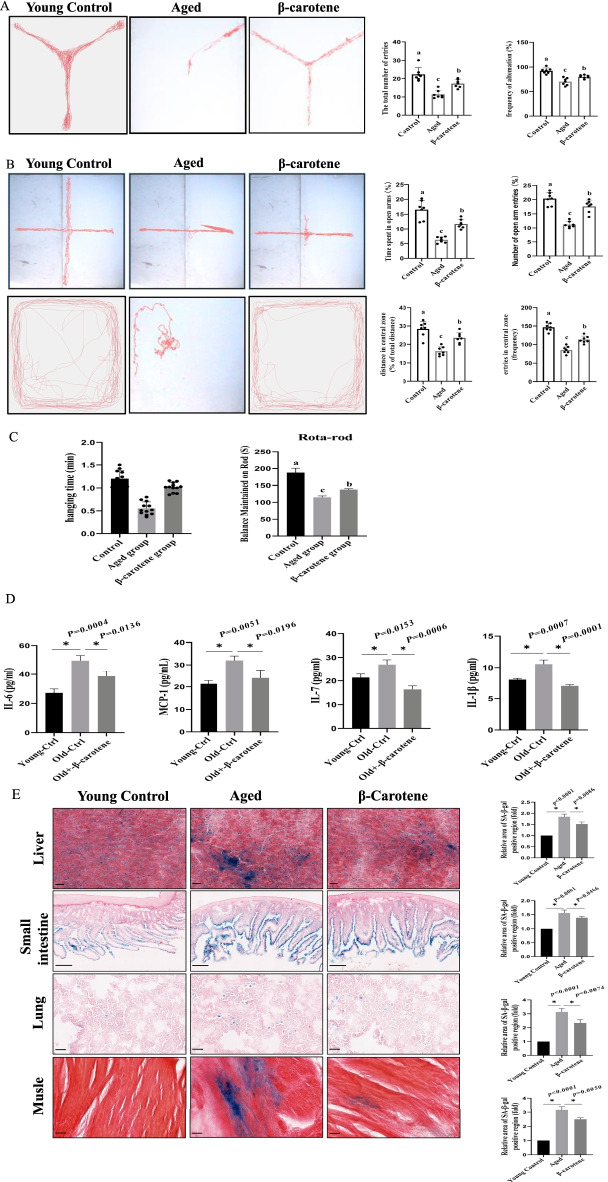

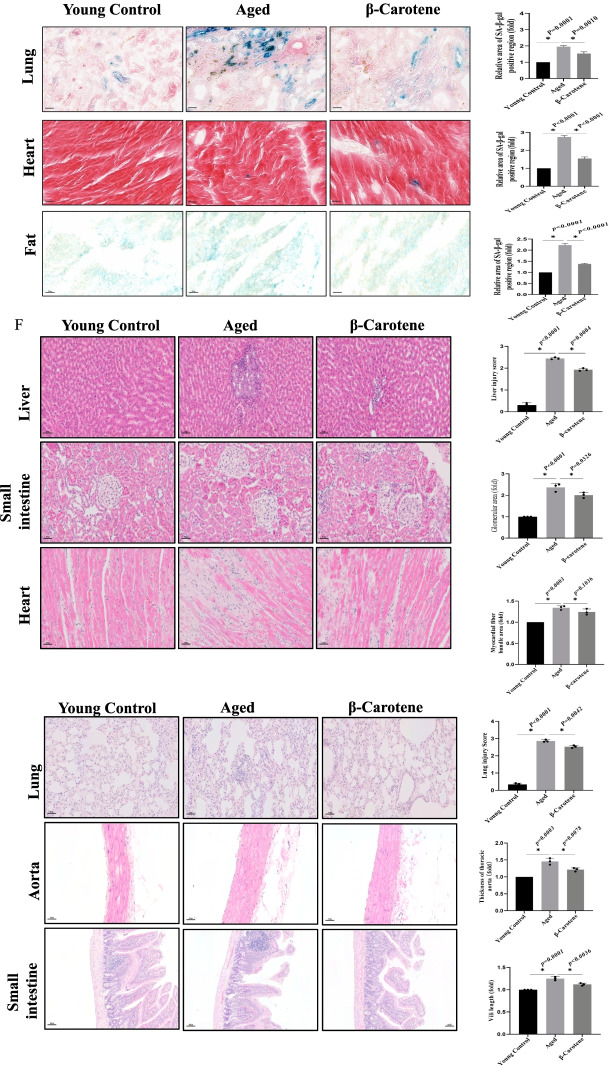

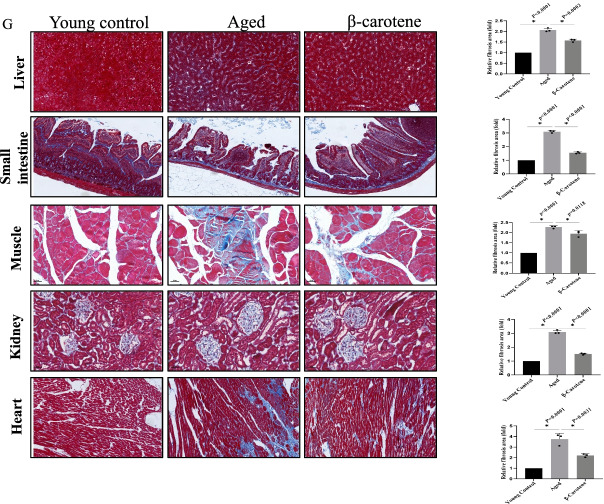
The effects of β-carotene on the behavior of the aged mice. **A** The effect of β-carotene on learning and cognitive behavior of mice determined by Y-maze. n = 5. **B** The effect of β-carotene on the anxiety by EPM and OFT analysis. n = 5. **C** β-Carotene improved the exercise ability of mice by hanging test and rotating rod test. **D** β-carotene reduced the inflammation. **E** The aging level of tissues and organs was evaluated by Sa-β-gal. **F** The tissues injury was reduced by β-carotene treatment. **G** The effect of β-carotene on tissue fibrosis

β-Carotene was tested for its effect on aged mice behavior from the perspectives of psychology and physiology. From the psychological angle, we tested the effect of β-carotene on mice learning and cognitive behavior using the Y-maze, which indicated that the aged mice treated with β-carotene demonstrated better learning and memory ability (Fig. 10A). The elevated Plus Maze (EPM) test found that β-carotene positively affected the level of anxiety in mice. β-Carotene treatment alleviated the anxiety of aged mice, and the same phenomenon was also found in further OFT experiments (Fig. 10B). From the physiological angle, we tested the effect of β-carotene on mice motorability. Both hanging test and rotating rod experiments demonstrated that β-carotene could improve the physical fitness of mice (Fig. 10C).

To further evaluate the anti-aging effect of β-carotene in vivo, we conducted the corresponding experiments on live mice. The results demonstrated that β-carotene significantly reduced the inflammation levels in vivo (Fig. 10D), Sa-β-gal staining demonstrated that the aging of tissues and organs was down-regulated (Fig. 10E), tissue injury was significantly reduced (Fig. 10F), and the level of tissue and organ fibrosis was down-regulated (Fibrosis is also an aging-related phenotype) (Fig. 10G). These basic observations indicate that β-carotene exhibited anti-aging effects in vivo.

## Discussion

Aging refers to the inevitable functional decline of an organism's organs at the end of its lifespan, accompanied by a lowered capacity to stabilize its internal environment and deal with stress. The deterioration of stem cells is considered the most important cause of aging; hence reversing or alleviating the aging of stem cells forms the main thrust of anti-aging research. In nature, β-carotene is widely distributed in vegetables and fruits. Animals and humans cannot synthesize β-carotene and must obtain it from their food. Previous studies have found that β-carotene has many biological functions in the human body, such as an anti-inflammatory, an antioxidant, and an anti-cancer [15–17]. So far, the effect of β-carotene on stem cell aging has not been reported. Therefore, in the current study, we evaluated the effect of β-carotene on the bioactivity of MSCs and found that β-carotene exhibited an anti-aging effect in vivo and in vitro.

Many factors contribute to aging, such as telomere shortening, oxidative stress, DNA damage, and cell cycle arrest [18, 19]. Recent studies have demonstrated that intestinal bacteria are involved in aging. However, at present, the establishment of an aging model in vitro is mainly through the following methods: (1) replicative aging; (2) oxidative stress-induced aging; (3) oncogene-induced aging, radiation-induced aging, and (4) drug-induced aging, etc. [20]. Among them, the H_2_O_2_-induced aging model is the most common. Therefore, we also successfully established the model of MSCs senescence using H_2_O_2_ in this work. Additionally, we also established a replicative aging model upon serial passaging.

Inflammation-related aging refers to the low-level, chronic, and systemic pro-inflammatory reaction state in natural aging [19]. Inflammation-related aging is characterized by a significant increase in inflammatory cytokines in the circulatory system, such as IL-1β, IL-6, and TNF-α [21]. The main cause of chronic inflammation in the aging process is the imbalance between pro-inflammatory and anti-inflammatory cytokines. In the current study, we found that β-carotene has obvious anti-inflammatory effects, which could significantly down-regulate the level of inflammatory cytokines (IL6 and TNFα). We further analyzed the potential mechanism by which β-carotene could inhibit inflammation, and the results showed that inflammation-related signaling pathways were significantly down-regulated by β-carotene treatment. In fact, previous studies have also shown that β-carotene shows the anti-inflammatory effect on various tissue and cell models [22, 23].

In the process of biological oxidative metabolism, cell may produce reactive oxygen species (ROS) and can be cleared by antioxidants in the body [24, 25, 28]. During aging, oxidative stress and ROS production significantly increase, and persistent oxidative stress exacerbates cell/tissue aging. In the current study, we found that β-carotene exhibits the anti-oxidative stress effect. Actually, many studies has reported that β-carotene displays the antioxidant function and role [26].

We have to ask How β-carotene relieves MSCs aging? Current research showed that β-carotene has anti-inflammatory and antioxidant effects, which may be potential anti-aging mechanisms of β-carotene, but this may not be the only mechanism. We further explore whether there are other potential mechanisms by which β-carotene could alleviate the aging of MSCs. After preliminary screening, we found that β-carotene could down regulate the expression of KAT7. Based on this, we speculate whether β-carotene exhibiting anti-aging effect may be through KAT7-p15^ink4b^ axis (at least partially).KAT7-p15^ink4b^ axis is a newly identified pathway involved in stem cell aging [14]. In the current work, we found that ectopic expression of KAT7 partially offset the anti-aging effect of β-carotene, indicating that β-carotene may achieve anti-aging effect byregulating KAT7-p15^ink4b^. In addition, to further confirm that β-carotene plays an anti-aging role through KAT7-p15 signaling axis [14]. We found that ectopic expression.

p15 partially offset β-carotene’s anti-aging effect. In addition, we also found that ectopic expression of KAT7 also up-regulated P15, indicating that p15 acts downstream of KAT7 in mediating cellular sensitivity. Of course, the anti-aging mechanism of β-carotene needs further in-depth research in the future, because MSC senescence is a very complex biological process [28–32].

Finally, we evaluated anti-aging effect of β-carotene through psychological and physiological perspectives in vivo, and results showed that β-carotene also exhibited anti-agingpotential in different tissues and organs in vivo.

## Conclusions

In conclusion, we found for the first time that β-carotene shows a good potential of anti-aging effect in vitro and in vivo, suggesting that β-carotene as a functional food has good potential application value in the field of anti-aging.

## Supplementary Information

**Additional file 1.** Primer sequence.

## Data Availability

The data from this study are available in this published article.

## Abbreviations

SIPS: Stress-induced premature senescence
RS: Replicative senescence
SA: Senescence-associated β-galactosidase
SD: Standard deviation
Bm-MSCs: Bone marrow-derived mesenchymal stem cells
