# Supplementary material for: Anti-aging effect of β-carotene through regulating the KAT7-P15 signaling axis, inflammation and oxidative stress process
Source: Cell Mol Biol Lett. 2022 Oct 8;27:86. doi: 10.1186/s11658-022-00389-7 (PMC9548120; doi:10.1186/s11658-022-00389-7)
Supplement: Supplementary file 1 — Additional file 1. Primer sequence. [file 11658_2022_389_MOESM1_ESM.docx]

IL-6, (F) 5′-GACAGCCACTCACCTCTTCA-3′,

1. 5′-TTCACCAGGCAAGTCTCCTC-3′;

TNF-α (F) 5′-GTCAGATCATCTTCTCGA ACC-3′,

1. 5′-CAGATAGATGGGCTCATACC-3′

Reference：

1. Fu, X., Gong, L. F., Wu, Y. F., Lin, Z., Jiang, B. J., Wu, L., & Yu, K. H. (2019). Urolithin A targets the PI3K/Akt/NF-κB pathways and prevents IL-1β-induced inflammatory response in human osteoarthritis: in vitro and in vivo studies. Food & function, 10(9), 6135-6146.
